# Pharm-MD; an open-label, randomized controlled, phase II study to evaluate the efficacy of a pharmacist-managed diabetes clinic in high-risk diabetes patients – study protocol for a randomized controlled trial

**DOI:** 10.1186/s13063-018-2836-8

**Published:** 2018-08-24

**Authors:** Alexandra Halalau, Daniel Shelden, Scott Keeney, Jaspreet Hehar

**Affiliations:** 1 Beaumont Health, Royal Oak, MI USA; 2 Oakland University William Beaumont School of Medicine, Royal Oak, MI USA

**Keywords:** High-risk diabetes mellitus, Morbidity, Mortality, Pharmacist, Hemoglobin A1c, Core measures

## Abstract

**Background:**

Millions of Americans are currently living with diabetes and approximately 1.5 million cases are being diagnosed each year. Diabetes is now the seventh leading cause of death in the United States. In addition, the economic burden of the disease has resulted in billions of dollars in health care costs. In spite of these investments, the United States lags behind other developed countries on diabetes life expectancy and disease-related deaths. The purpose of this study is to assess the impact of a pharmacist-managed diabetes clinic (PMDC) model on diabetes core measures. Our hypothesis is that a PMDC would have a significant positive impact on the diabetes measures and will result in higher-quality care at a lower price.

**Methods:**

This study is a randomized, open-label, controlled, parallel-group trial which will be conducted in the outpatient clinic at Beaumont Hospital, Royal Oak, Michigan. Patients will be randomly assigned to one of two groups: standard of care (SOC) or standard of care plus PMDC (SOC + PMDC). Included in the study will be patients older than 18 years of age with a diagnosis of type 2 diabetes mellitus and a hemoglobin A1c ≥ 9%, who are established with a primary care resident and who have not been seen in the PMDC within the last 3 months. The primary outcome is the change in hemoglobin A1c, measured at 6 and 12 months. Secondary outcomes include the impact on all diabetes core measures, patient quality of life, harms, and cost impact related to the intervention.

**Discussion:**

If the results of this trial are consistent with the previous retrospective analysis that a pharmacy clinic has a significant impact in controlling hemoglobin A1c levels as well as other diabetes core measures to improve clinical outcomes, it will constitute a scaffold for a future multicenter, randomized controlled trial. In addition, these results may influence future diabetes guidelines, leading to the inclusion of a PMDC as the standard of care. The impact of these results on the economic burden, life expectancy, and diabetes-related deaths are needed and have yet to be studied.

**Trial registration:**

ClinicalTrials.gov, ID: NCT03377127.

Protocol version: registered on 10 February 2018; version #1.

**Electronic supplementary material:**

The online version of this article (10.1186/s13063-018-2836-8) contains supplementary material, which is available to authorized users.

## Background

### Background and rationale

Over the past 20 years, the number of adults with diabetes has doubled in the United States (US) [1]. According to the Centers for Disease Control (CDC), diabetes mellitus (DM) affected 30.2 million adults in 2015, with 1.6 million new cases of diabetes diagnosed in adult Americans. DM was the seventh leading cause of death in 2015 in the US [2]. Additionally, DM is associated with significant morbidity, leading to an estimated cost of US$245 billion in 2012. Medical costs are 2.3 times higher for patients with diabetes when adjusting for age and gender [2]. In addition to costs to the health care system, insulin prices have nearly tripled between 2002 and 2013, often directly impacting patients [3]. People with diabetes are twice as likely to have heart disease or stroke compared to those without diabetes [4]. Diabetes is also the leading cause of kidney failure [5]. For patients over 40 years of age who have severe non-proliferative diabetic retinopathy or with proliferative retinopathy, the prevalence of visual impairment is 48% [6]. Patients with diabetes have a life expectancy 6 years shorter than those who do not have diabetes. An estimated 40% of this difference is due to non-vascular conditions such as several cancers, infectious diseases, external causes, intentional self-harm, and degenerative disorders [7].

In 2016, the Association of American Medical Colleges estimated that the shortage of physicians in the United States would be between 61,700 and 94,700 by 2025 [8]. The data suggest that, while the number of patients with diabetes continues to increase, the physician shortage is becoming more prominent. According to the Bureau of Labor Statistics, nurse practitioners and physician assistants are expected to show a job growth of 31% and 37%, respectively, over the next 10 years [9, 10]. The goal of using physician extenders is to mitigate cost in a safe fashion while providing adequate high-quality medical care to patients. Data identified in our literature review suggest that pharmacists are a potential underutilized resource [11–17]. Clinical pharmacists can provide patient care that optimizes medication management, health, wellness, and disease prevention [18]. Utilizing a physician-pharmacist collaborative model has shown benefit across a variety of chronic conditions [19]. In an analysis of pharmacist-managed diabetes care clinics, Morello et al. noted increased rates of screening for comorbid conditions associated with diabetes as well as a higher rate of meeting treatment goals when compared to prior to their intervention [11]. Anaya et al. evaluated a similar model noting improvement in glycemic control while identifying the added benefit of decreased hospitalization and emergency center (EC) visits in their intervention group [12]. Kelly and Rodgers addressed the efficacy of a pharmacist-physician model in a prospective analysis, again noting improved hemoglobin A1c (HbA1c) in the pharmacist-managed arm [13]. Choe et al., Leal et al., and Davidson et al. found similar advantages associated with a pharmacist-managed diabetes model [14–16].

Diabetic core measures are standards set forth by the American Diabetes Association (ADA) for the treatment of this chronic condition. With the development of newer medications and treatment approaches, the ADA suggests annual updates and recommendations based on the medical literature. Thus, the periodic evaluation of HbA1c, low-density lipoprotein (LDL) cholesterol, microalbuminuria, and retinal examination became the standard of care. The identification of intermediate metrics, such as control of blood pressure, HbA1c, and LD- cholesterol and the association between improved control of these factors and clinical outcomes including cardiovascular events, microvascular complications, and mortality, has been proven. Previous studies demonstrated that for each 1% reduction in HbA1c, there was a corresponding 14% reduction in myocardial infarction, 12% reduction in stroke, and a 37% reduction of microvascular complications [4].

Based on the knowledge obtained from prior studies [20], we looked at ways to potentially improve the care provided to our patients, and a pharmacist-managed diabetes clinic (PMDC) was created. The uniqueness of our PMDC, which differentiated it from prior studies, was the focus of the educational visits on patient-identified goals and barriers to attaining better diabetes management. In addition, the pharmacists were able to verify the medication list, simplify and check for safety and interactions between medications and also perform dose adjustments supported through their collaborative agreement with our clinic. Our preliminary retrospective data showed that the PMDC patients had a decrease at 6 months in HbA1c of 3.2% versus 1.2% in the standard care cohort (*p* = 0.044) [20]. Our study aims to prospectively assess the impact of a PMDC model on the diabetes core measures in patients with high-risk diabetes mellitus.

### Study hypothesis

Our hypothesis is that a pharmacist-managed diabetes clinic focusing on patient-identified diabetes management gaps and goals would have a significant positive impact on HbA1c and on diabetes core measures and will result in a higher quality of care at a lower price.

### Trial design

This is a randomized, open-label, controlled, parallel-group trial of a pharmacist-managed diabetes clinic in high-risk diabetes patients, with a 1:1 allocation to either standard of care (SOC) or standard of care plus pharmacy managed diabetes clinic (SOC + PMDC) with a 6-month and 12-month follow-up.

## Methods

### Study setting

The study will be conducted in the outpatient clinic at Beaumont Hospital, Royal Oak, Michigan. The outpatient clinic is a resident clinic that delivers medical care to over 920 patients with diabetes mellitus. The clinic is based on campus at Beaumont Hospital. The patients are assigned to 60 internal medicine residents and 16 medicine-pediatric residents. The residents function under the direct supervision of a board-certified internal medicine and attending physician. Patient care is always discussed with, and approved by, the attending physician. Potential subjects with high-risk diabetes mellitus will be identified through weekly reports and from the daily schedule and will be recruited from this pool of patients exclusively.

### Eligibility criteria

#### Inclusion criteria

The target population will be comprised of high-risk diabetes mellitus type 2 patients (HbA1c ≥ 9%) that are not currently enrolled in the PMDC. In order for the patient to be eligible for enrollment, they will have to be established with a primary care internal medicine or medicine-pediatric resident and will have to make a diagnosis of diabetes mellitus type 2. The patients will be screened based on the diabetic registry in the electronic medical record (EMR).

#### Exclusion criteria

Patients will be excluded if they have been seen by the PMDC within the past 3 months. This time period was chosen to mitigate the risk for the potential confounder of a PMDC visit directly affecting the HbA1c. By selecting this time period, we aim to maintain a larger pool of patients to select from than we would otherwise if we completely excluded patients who had previously been seen in the PMDC. Patients will be excluded if they are under 18 years of age or over 75 years of age. Patients will also be excluded if they are documented as having type 1 diabetes mellitus.

### Intervention

The patients will be enrolled over a 6-month period and will be randomly assigned to the control group (standard of care (SOC)) or the intervention group (SOC + PMDC visits). The PMDC is a pharmacist-led clinic that has been functioning in our clinic since January 2015 and is considered an available resource.

**Fig. 1:**
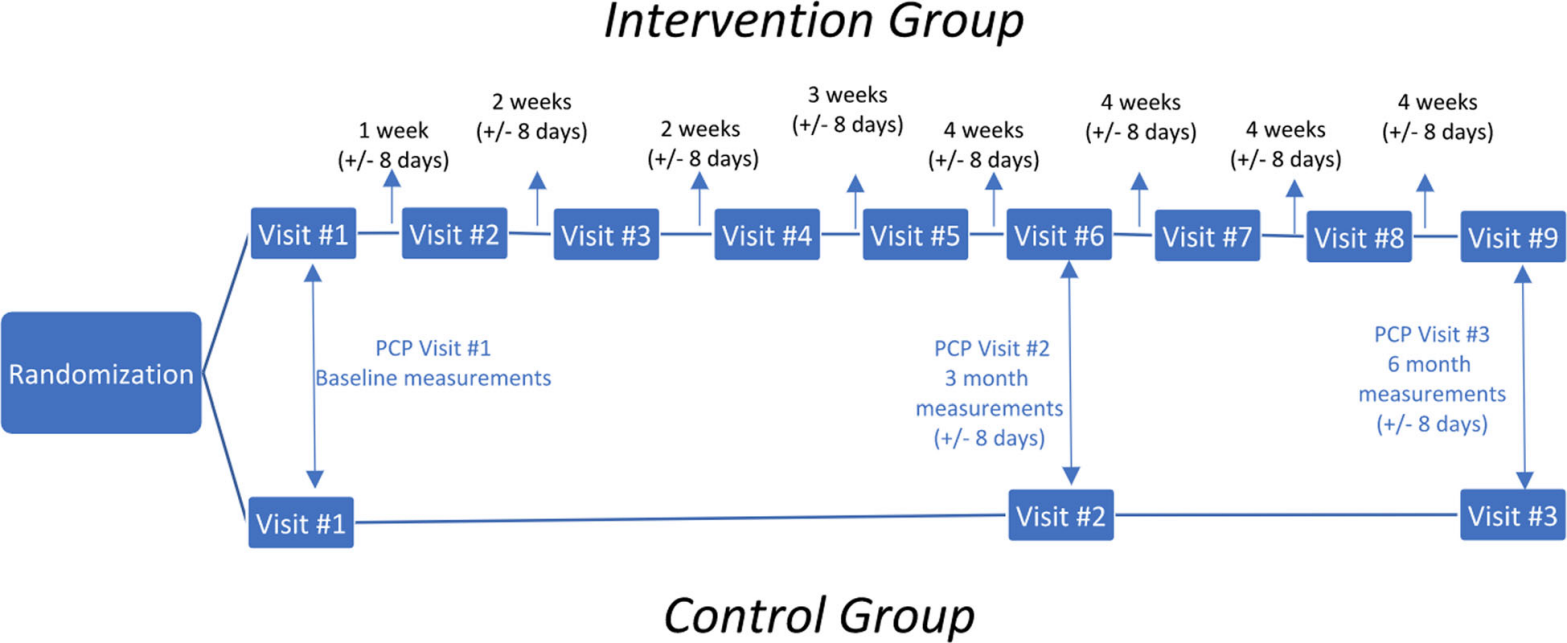
Patient intervention timeline

The intervention group patients will be managed by their assigned primary care physicians (PCPs) per the standard of care and will have scheduled six extra face-to-face visits with the pharmacists for the 6-month duration of the intervention (Fig. 1). The PMDC visits will be scheduled more frequently in the first 3 months of the intervention to ensure that patients’ engagement and provide enough opportunities and time to address all the patients’ goals and concerns. The PMDC visit encounters will focus on patient-identified goals for the management of their diabetes. The initial visit in the PMDC will be 60–90 min with follow-up visits lasting 30–45 min. Patients will be asked to describe their own gaps in knowledge and to identify their own management goals. Identification of knowledge gaps will allow targeted patient education to close those gaps. Other educational opportunities will potentially include diabetes mellitus pathophysiology, blood glucose goals, HbA1c goals, management of hyperglycemic and hypoglycemic episodes, review of medications, and counseling regarding diet and exercise. Pharmacists have the discretion to make medication adjustments and initiate new medications pertinent to the management of diabetic comorbidities. The model is a collaborative practice agreement between the pharmacist and the PCP.

The control group patients will be managed by their assigned PCPs per the standard of care. Management per the standard of care includes referrals to ophthalmology for a dilated-pupil eye examination, nephrology for nephropathy management, cardiology for macrovascular complications management, neurology for neuropathy or neurological complications, diabetic education, laboratory studies, and vaccinations and will be ordered or performed at the discretion of each patient’s PCP.

We do not foresee any harm resulting from our intervention. These patients can only be enrolled in the PMDC through the study if they are randomized to the SOC + PMDC group. The patients will be counseled through the information sheet that if they were randomized to the SOC group and would wish to be enrolled in the PMDC, they can do so only after they complete the 12 months in the study.

In order to improve adherence with the intervention protocol and minimize overall attrition, the patients will receive monetary compensation for the visits built in the study timeline. Each patient in the SOC + PMDC group will receive a US$15 gift card per visit for the nine visits over the 6 months. Each patient in the SOC group will receive a US$15 gift card per visit for the three visits considered standard of care. The gift cards will be handed at each visit, after the visit completion. If a patient has a rescheduled visit outside of the window of ± 8 days from the initial scheduled visit, no gift card will be given. If patients cancel and reschedule any of the follow-up visits, the registration department will send an email to the research assistant to notify her of the changed appointment. In order to do this, the registration department will be trained regarding the study protocol and the required follow-up scheduled visits. The patients will be labeled in the EMR under patient notes as being part of the study. Their group allocation will not be revealed in the event that the data collectors, outcome assessors or the biostatistician will have access to their medical record. If a patient does not show up for their scheduled appointment, the research assistant will call the patient in an attempt to reschedule within the time frame of 8 days. The patient will be called three times. The research assistant will document and keep track of the phone calls.

Outside the intervention, the participants in both groups will be treated identically. They will participate in the standard of care visits at baseline, 3 months, and at 6 months. These visits (visit 1, 6, and 9 in the SOC + PMDC group and visit 1, 2, and 3 in the SOC group) will be provided by each patient’s PCP (Fig. 1).

### Outcomes

The primary outcome for this study is the change in HbA1c between the groups (intervention versus control), measured at 6 months.

**Fig. 2:**
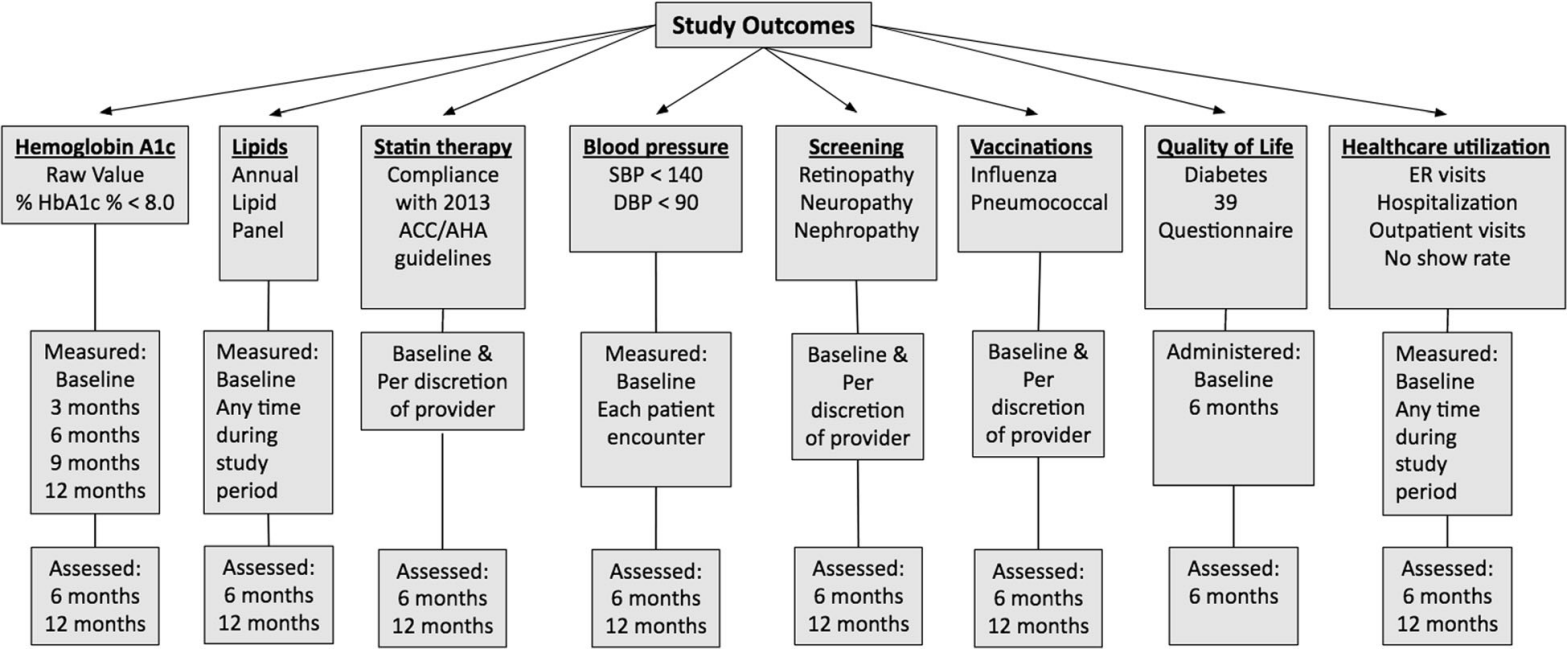
Measurement of study outcomes, and assessment timelines

Secondary outcomes for this study are related to the impact of the PMDC on all diabetes core measures, patient quality of life (QOL), and harms and cost-effectiveness related to the intervention (Fig. 2). Secondary outcomes for this study include the following:

- HbA1c change between the control and intervention groups at 12 months after randomization
- Percentage of patients achieving a HbA1c measurement of less than 8% at 6 months and 12 months
- The change in HbA1c between 6 months and 12 months after randomization
- Achievement of annual lipid panel testing
- Compliance with statin therapy per the 2013 American College of Cardiology / American Heart Association guidelines
- Blood pressure goal of less than 140/90 mmHg at the end of the trial period
- Compliance with recommended diabetic screening: annual retinopathy examination via dilated-pupil retinal examination, annual nephropathy evaluation with urine microalbumin testing, and annual neuropathy evaluation with lower-extremity monofilament testing
- Compliance with recommended vaccinations including annual influenza vaccination and pneumococcal vaccination
- QOL assessment via the Diabetes-39 questionnaire administered at baseline and at 6 months
- Number of EC visits related to hyperglycemia or hypoglycemia and all EC visits measured at 6 and 12 months
- The overall quantity of total inpatient visits
- The overall number of total outpatient visits
- The “no-show” rate

### Participant timeline

**Fig. 3:**
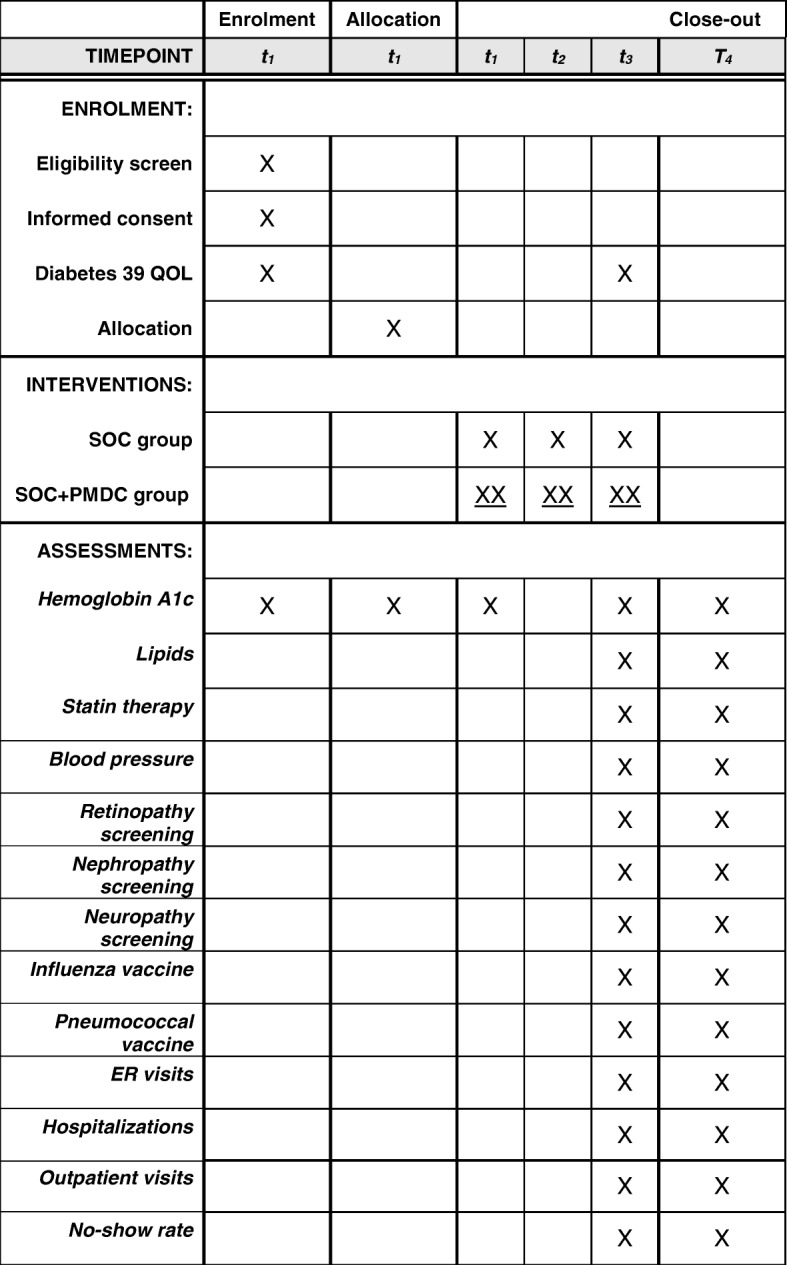
Schedule of enrollment, interventions, and assessments

Once enrolled in the study, at the time of their PCP visit, all the patients will complete the Diabetes-39 survey, the diabetes mellitus length of diagnosis will be confirmed and then they will complete the visit with their PCP. After their PCP visit, each patient will be given a schedule with their subsequent visit dates and times during the 6-month intervention time, as follows: the SOC patients will be scheduled to follow-up with their PCP in 3 and 6 months and the SOC + PMDC patients will be scheduled for the first visit with the PMDC as per Fig. 1 and return for the two follow-up visits with their PCP in 3 and 6 months (SOC appointment card). After the completion of the first PMDC visit, the patients will receive an appointment card that will have the dates and times for the following five PMDC visits (pharmacy appointment card). All the patients will have to complete the Diabetes-39 survey at the third PCP visit Fig. 3. All the follow-up visits will have a window of 8 days before and after the appointment to allow patients the flexibility to reschedule their appointments in case they are not able to attend the initial scheduled appointment. After the enrollment, the research assistant will make sure that the follow-up visits are scheduled per study protocol and will give the patients the appointment cards filled out with the dates and times. At the time of the first PMDC visit, the pharmacist will be responsible for giving the patients their appointment card with the schedule of the next five PMDC visits.

### Sample size

Assigning an allocation of 1:1 that will result in equal sample sizes, we are expecting a mean difference of change in HbA1c of 1% between the two groups (intervention versus control) with standard deviation (SD) of 1.5%. The sample size was estimated to be 36 per arm or a total of 72 with 80% power at a *p* < 0.05 significance. Adding 20% for attrition, the final sample size was estimated at 86 patients. We considered a HbA1c difference of 1% as being clinically significant in long-term reduction of stroke, myocardial infarction, and microvascular complications [3]. PASS 15 Power Analysis and Sample Size Software (2017). NCSS, LLC. Kaysville, UT, USA, ncss.com/software/pass was used for the sample size calculation.

### Recruitment

The recruitment is planned to take place over 6 months.

The patients will be identified via the diabetic registry through business office software and through the daily clinic schedule from the EMR. The patients with HbA1c greater or equal to 9% and overdue for their PCP follow-up diabetes visit, identified through the diabetes registry, will be called to schedule an appointment with their PCP. They will be approached at the time of the clinic visit for enrollment in the study. The patients will also be identified from the daily schedule through electronic reports from the EPIC EMR. Daily reports will be run for the next day to identify the patients with diabetes and HbA1c greater or equal to 9% that have an appointment with our clinic. Manual chart review will be performed to confirm the eligibility criteria based on HbA1c greater or equal than 9% and not actively enrolled in the PMDC clinic. Recruitment letters will be mailed to all the patients found eligible to be enrolled in the study based on their HbA1c level (Additional file 1) The patients will be directed to call the research assistant and they will be helped to schedule a PCP appointment if they are overdue. We considered overdue appointment for patients with a HbA1c greater or equal than 9% if they have not seen their PCP in over 3 months. If the patients had a recent PCP appointment and, therefore, are not overdue, but are interested to be enrolled in the study, the research assistant will help them schedule their next PCP visit and will advise them that they will be enrolled at that visit. The research assistant will keep record of these patients and their follow-up PCP appointment date and time.

### Allocation

Subjects will be allocated to SOC + PMDC or SOC (1:1) using the method of blocked randomization with alternating size blocks of 4, to ensure the same number of patients in each arm periodically but keeping the next allocation blinded. Patients will have an equal probability of being randomized to the treatment or the control arm. The randomization scheme will be computer generated. The randomization scheme and the study envelopes with the name of the study, an ID number for that patient and the arm to which they are randomized, based on the randomization scheme, will be prepared by a biostatistician who will not be involved in the study. The envelopes with each assigned group will be closed and opaque and will be given to the research assistant. Efforts will be made to maintain the allocation sequence concealed. The opaque envelopes that will contain the group allocation will be kept in a locked box in the clinic. The research assistant will be the only person that will have access to the box. Once the eligible patients present to their PCP visit, the research assistant will provide the information sheet to the patients and enroll them into the study. The research assistant will fill out a receipt of information sheet that will document the date and the time the patient was enrolled in the study. She will then give the patients the next sequential available envelope with the assigned group protocol (intervention or control group), per the randomization scheme. The envelopes will contain a notecard with the subject ID and the assigned name of the group (SOC or SOC + PMDC). A copy of the notecard will be kept with the patients’ study documents to ensure the accuracy of the patient allocation. The research assistant will be permanently under the supervision of a medically trained principal investigator. The research assistant will aid in scheduling the patients for follow-up in accordance with their assigned treatment arm. To ensure allocation concealment, the research assistant will not be aware of the randomization scheme and will not be able to see the content of the envelopes until after the patient allocation.

### Blinding

As this is an open-label study, the patients and the physicians will not be blinded to the intervention. However, the data collectors, data analysts, and outcome assessors will be unaware of the patients allocation. Adequate measures will be taken throughout the entire duration of the trial to minimize any possible ascertainment bias in this study. These measures will include efforts to maintain blinding of the outcome assessors to avoid observer bias and to ensure blinding of the data collectors and data analysts by coding the participants in the two groups until the statistical analysis is completed, to also avoid selective reporting bias. The groups will be labeled with non-identifying terms (such as A and B) by the research assistant at the time of randomization. Only the research assistant will be aware of the real allocation. She will enter the enrolled patients in a spreadsheet within the shareable online Sharepoint database and assign each patient a letter (A or B) based on their group allocation, in the SOC + PMDC or in the SOC group. The group number assigned will not be revealed to any study personnel until the final statistical analysis has been completed. The biostatistician will also be blinded, to not know the equivalence of the groups (A and B) with the SOC + PMDC or SOC group.

### Data collection

A predetermined spreadsheet with the baseline characteristics variables will be used to collect the data and it will include: HbA1c, age, gender, race, length of time with diabetes, Body Mass Index (BMI), systolic blood pressure (SBP), diastolic blood pressure (DBP), creatinine, estimated glomerular filtration rate, microalbumin, total cholesterol, LDL-cholesterol, high-density lipoprotein cholesterol (HDL)-cholesterol, triglycerides, tobacco-use status, neuropathy, retinopathy, nephropathy, macrovascular complications, oral medications only, insulin combinations, and Diabetes-39 survey.

The majority of the outcome data will be collected at 6 and 12 months after starting the intervention. This will include HbA1c value as both a continuous variable and with the “goal of ≤ 8%.” Also, lipid panel, blood pressure management (goal of < 140/90 mmHg), compliance with recommended diabetes mellitus screening (annual retinopathy, annual nephropathy, and annual neuropathy evaluation), immunizations (influenza and pneumonia vaccines), QOL (Diabetes-39 survey), number of overall EC visits and EC visits for hyperglycemia or hypoglycemia, overall inpatient and outpatient visits, and “no-show” rate.

The study personnel will include investigators, research assistants, and research nurse managers. They will be trained to enroll patients and elicit information and collect questionnaires from patients in a uniform manner.

The Diabetes-39 questionnaire captures many subjective aspects of QOL. This questionnaire focuses on an individual’s own views of their well-being.

In order to promote participant retention and complete follow-up, the following will be taken into account: regarding surveys, only data that are necessary to answer research questions will be asked. Participants will be asked to provide their cell phone number, to indicate best time to contact them and whether messages can be left. Questionnaires are intended to be filled during the PCP visits (1 and 9 for the intervention group and 1 and 3 for the standard of care group) or may be completed by telephone or be sent via mail or email to make it more convenient for the patients. For the working populations; suitable visit times will be scheduled (early morning/afternoons). The demographic status of participants will be regularly updated at the time of their visit. An online or manual tracking system will be used to schedule visits. Visits will be scheduled at the same time each time to reduce no shows. Reminder messages and phone calls about appointments will be sent. Every attempt will be made to identify patients lost to follow-up and make an effort to locate them.

All data will be entered electronically to a locked/secured database. Access to the data will be limited to research personnel only. Administrator access rights will be limited to the principal investigators. Activity is regulated using identification codes and passwords. Study forms/questionnaires will be printed and completed forms will be kept in a locked file cabinet located in the research assistant office. Names will be replaced with encoded identifiers, with the key kept in a locked file cabinet. The data with encoded identifiers will be given to the biostatistician for the final data analysis. Data will be maintained in storage for a period of 11 years after the study is completed. Electronic backup of the data will occur every week.

### Statistical methods

The primary analysis of all primary and secondary outcomes will be on an intention-to-treat (ITT) basis. A secondary “per-protocol” analysis will be completed including only patients adhering to the study protocol and not crossing over to the other group. All missing data will be replaced with substitutions or imputations. The last observation carried forward will be used to replace the missing data. The ITT analysis will include all patients randomized regardless of how many PMDC or SOC visits were attended. The per-protocol analysis will only include patients that have met at least 67% of their visits. Any PMDC patient that has missed more than two visits will not be included in this per-protocol analysis. We may do multivariable analyses to adjust for any baseline differences found between the two randomization arms. All analyses will be completed under the guidance of the Beaumont Research Institute Biostatistics Department.

Descriptive statistics will be given for all data collected. Categorical variables will be reported as counts and % frequencies. Continuous variables will be reported as means ± the SD followed by the median then minimum to maximum or median (25th, 75th percentiles) then minimum to maximum dependent on the normality of the data. The two randomization groups will be examined for all variables. Categorical variables will be examined using Pearson’s chi-square tests where appropriate (expected frequency > 5); otherwise, Fisher’s exact tests will be used. Continuous variables will be examined for normality. Normally distributed variables will be examined using two-sided *t* tests. Non-normally distributed variables will be examined using non-parametric Wilcoxon rank tests.

The primary outcome will be examined between the two randomization arms with either a *t* test or a Wilcoxon rank test dependent on the normality of the changes. Within each arm, the paired difference between baseline and 6 months will be examined with a sign test to see if the change is different than 0.

Secondary outcomes for this study will be examined as follows: percentage of patients achieving a HbA1c measurement of less than 8% at 6 months and then again at 12 months will be examined with a Pearson’s chi-square test if appropriate (expected frequency > 5), otherwise Fisher’s exact tests will be used. The change in HbA1c at 12 months after randomization will be examined between the two randomization arms with either a *t* test or a Wilcoxon rank test dependent on the normality of the changes. Within each arm, the paired difference between baseline and 6 months and baseline and 12 months will be examined with a sign test to see if the change is different from 0. The achievement of annual lipid panel testing, compliance with statin therapy per the 2013 American College of Cardiology / American Heart Association guidelines, reaching blood pressure goal of less than 140/90 at 12 months, compliance with recommended diabetic screening (i.e., annual retinopathy examination via dilated-pupil retinal examination, annual nephropathy evaluation with urine microalbumin testing, and annual neuropathy evaluation with lower-extremity monofilament testing), and compliance with recommended vaccinations including annual influenza vaccination and pneumococcal vaccination will all be examined with Pearson’s chi-square tests if appropriate (expected frequency > 5), otherwise Fisher’s exact tests will be used. The QOL assessment via the Diabetes-39 questionnaire administered at baseline and at the conclusion of the intervention period (6 months) will be examined for the total score between the two randomization arms with either a *t* test or a Wilcoxon rank test dependent on the normality of the changes. Number of EC visits related to hyperglycemia or hypoglycemia at 6 and 12 months, overall quantity of total inpatient and outpatient visits, all EC visits and the “no-show” rate will be examined with Pearson’s chi-square tests if appropriate (expected frequency > 5); otherwise, Fisher’s exact tests will be used.

### Monitoring

#### Data monitoring

Harms associated with this intervention are defined as hypoglycemic episodes that resulted in EC evaluation or hospital admission and will be evaluated as secondary outcomes. There are no other possible harms associated with the intervention. Therefore, a Data Monitoring Committee (DMC) is not needed.

Given the small sample size, we do not plan to execute any interim analysis. Therefore, the study will not be considered at any point to be terminated based on harm or benefit.

#### Bias

One potential significant source of bias identified is attrition. To mitigate attrition, patients will receive a small stipend for each of their visits, per the study protocol, to help offset potential costs associated with their transportation.

## Discussion

The main operational concern related to this trial is the enrollment. We anticipate that we might have a slow enrollment rate secondary to limited resources. We plan to have weekly spread sheets to keep track of the amount of patients eligible for the study, patients approached and patients consented. A spread sheet with all the approached patients and reasons for enrollment refusal will be kept and analyzed for potential actions to decrease the enrollment barriers. These spread sheets will be reviewed every months by the principal investigator. More so, the principal investigator will have monthly meetings with the research assistant and with the research nurse manager to discuss any enrollment problems or any other issues that arise during the study period. We will also create a study brochure to be distributed by the residents to the eligible patients. The brochure will contain information about the study and the intervention and also the contact information of the research assistant for the patients that are interested in enrolling.

### Trial status

At the time of this manuscript submission for publication, patient recruitment had not yet started.

## Additional files

Additional file 1: Institutional Review Board (IRB)-approved informed consent; outcome letter with IRB approval; grant award letter; pharmacy visit template; standard of care (SOC) appointment card; standard of care + pharmacist-managed diabetes clinic (SOC + PMDC) appointment card; pharmacy appointment card; Diabetes-39 questionnaire. (ZIP 443 kb)

Additional file 2: Standard Protocol Items: Recommendations for Interventional Trials (SPIRIT) 2013 Checklist: recommended items to address in a clinical trial protocol and related documents* (DOC 121 kb)

## Abbreviations

ADA: American Diabetes Association
BMI: Body Mass Index
CDC: Centers for Disease Control
DBP: Diastolic blood pressure
DM: Diabetes mellitus
EC: Emergency center
eGFR: Estimated glomerular filtration rate
HbA1c: Hemoglobin A1c
HDL: High-density lipoprotein
IRB: Institutional Review Board
ITT: Intention-to-treat
LDL: Low-density lipoprotein
PCP: Primary care physician
PMDC: Pharmacist-managed diabetes clinic
QOL: Quality of life
SBP: Systolic blood pressure
SOC: Standard of care
US: United States
