# Supplementary material for: Pharm-MD; an open-label, randomized controlled, phase II study to evaluate the efficacy of a pharmacist-managed diabetes clinic in high-risk diabetes patients – study protocol for a randomized controlled trial
Source: Trials. 2018 Aug 24;19:458. doi: 10.1186/s13063-018-2836-8 (PMC6109355; doi:10.1186/s13063-018-2836-8)
Supplement: Supplementary file 1 — Institutional Review Board (IRB)-approved informed consent; outcome letter with IRB approval; grant award letter; pharmacy visit template; standard of care (SOC) appointment card; standard of care + pharmacist-managed diabetes clinic (SOC + PMDC) appointment card; pharmacy appointment card; Diabetes-39 questionnaire. (ZIP 443 kb) [file 13063_2018_2836_MOESM1_ESM.zip › Diabetes 39 surveyR1.pdf]

Subject ID:_____
Date: _____

Diabetes-39

Quality of Life Questionnaire – Baseline / 6 Months

IRB# 2017-494 - Study Name_____

A person's quality of life is affected by many things. These things might include health, the opportunity for recreation and holidays, friends and family, work and the hassles and inconveniences of diabetes. This questionnaire is designed to help us learn more about what affects the quality of life of people with diabetes.

How to complete the questionnaire.

- For each of the following questions we want to know how much your quality of life has been affected. Please circle a number which you think best describes how your quality of life has been affected in the past month.
- If you have any questions about how to complete the questionnaire, please ask the research coordinator

During the <u>past month</u> how much was the quality of <u>your</u> life affected by:

1. Your daily medication for your diabetes

Not affected At all	1	2	3	4	5	6	7	Extremely Affected

2. Worries about money matters

Not affected At all	1	2	3	4	5	6	7	Extremely Affected

3. Limited energy levels

Not affected At all	1	2	3	4	5	6	7	Extremely Affected

4. Following your doctor's prescribed treatment plan for diabetes

Not affected At all	1	2	3	4	5	6	7	Extremely Affected

5. Food restrictions to control your diabetes

Not affected At all	1	2	3	4	5	6	7	Extremely Affected

Subject ID:_____
Date: _____

6. Concerns about your future

Not affected								Extremely
At all	1	2	3	4	5	6	7	Affected

7. Other health problems besides diabetes

Not affected								Extremely
At all	1	2	3	4	5	6	7	Affected

8. Stress or pressure in your life

Not affected								Extremely
At all	1	2	3	4	5	6	7	Affected

During the <u>past month</u> how much was the quality of <u>your</u> life affected by:

9. Feelings of weakness

Not affected								Extremely
At all	1	2	3	4	5	6	7	Affected

10. Restrictions on how far you can walk

Not affected								Extremely
At all	1	2	3	4	5	6	7	Affected

11. Any daily exercises for your diabetes

Not affected								Extremely
At all	1	2	3	4	5	6	7	Affected

12. Loss or blurring of vision

Not affected								Extremely
At all	1	2	3	4	5	6	7	Affected

13. Not being able to do what you want to do

Not affected								Extremely
At all	1	2	3	4	5	6	7	Affected

Subject ID:_____
Date: _____

14. Having diabetes

Not affected At all	1	2	3	4	5	6	7	Extremely Affected

15. Losing control of your blood sugar levels

Not affected At all	1	2	3	4	5	6	7	Extremely Affected

16. Other illnesses besides diabetes

Not affected At all	1	2	3	4	5	6	7	Extremely Affected

During the <u>past month</u> how much was the quality of <u>your</u> life affected by:

17. Testing your blood sugar levels

Not affected At all	1	2	3	4	5	6	7	Extremely Affected

18. The time required to control your diabetes

Not affected At all	1	2	3	4	5	6	7	Extremely Affected

19. The restrictions your diabetes places on your family and friends

Not affected At all	1	2	3	4	5	6	7	Extremely Affected

20. Being embarrassed because you have diabetes

Not affected At all	1	2	3	4	5	6	7	Extremely Affected

21. Diabetes interfering with your sex life

Not affected At all	1	2	3	4	5	6	7	Extremely Affected

Subject ID:_____
Date: _____

22. Feeling depressed or low

Not affected At all	1	2	3	4	5	6	7	Extremely Affected

23. Problems with sexual functioning

Not affected At all	1	2	3	4	5	6	7	Extremely Affected

24. Getting your diabetes well controlled

Not affected At all	1	2	3	4	5	6	7	Extremely Affected

During the <u>past month</u> how much was the quality of <u>your</u> life affected by:

25. Complications from your diabetes

Not affected At all	1	2	3	4	5	6	7	Extremely Affected

26. Doing things that your family and friends don't do

Not affected At all	1	2	3	4	5	6	7	Extremely Affected

27. Keeping a record of your blood sugar levels

Not affected At all	1	2	3	4	5	6	7	Extremely Affected

28. The need to eat at regular intervals

Not affected At all	1	2	3	4	5	6	7	Extremely Affected

29. Not being able to do housework or other jobs around the house

Not affected At all	1	2	3	4	5	6	7	Extremely Affected

Subject ID:_____
Date: _____

30. A decreased interest in sex

Not affected At all	1	2	3	4	5	6	7	Extremely Affected

31. Having to organize your daily life around diabetes

Not affected At all	1	2	3	4	5	6	7	Extremely Affected

32. Needing to rest often

Not affected At all	1	2	3	4	5	6	7	Extremely Affected

During the <u>past month</u> how much was the quality of <u>your</u> life affected by:

33. Problems in climbing stairs or walking up steps

Not affected At all	1	2	3	4	5	6	7	Extremely Affected

34. Having trouble caring for yourself (dressing, bathing, or using the toilet)

Not affected At all	1	2	3	4	5	6	7	Extremely Affected

35. Restless sleep

Not affected At all	1	2	3	4	5	6	7	Extremely Affected

36. Walking more slowly than others

Not affected At all	1	2	3	4	5	6	7	Extremely Affected

37. Being identified as a diabetic

Not affected At all	1	2	3	4	5	6	7	Extremely Affected

Subject ID:_____
Date: _____

38. Having diabetes interfere with your family life

Not affected At all	1	2	3	4	5	6	7	Extremely Affected

39. Diabetes in general

Not affected At all	1	2	3	4	5	6	7	Extremely Affected

OVERALL RATINGS

1. Please circle below to indicate your overall rating of quality of life

Not affected At all	1	2	3	4	5	6	7	Extremely Affected

2. Please circle below to show how severe you think your diabetes is

Not affected At all	1	2	3	4	5	6	7	Extremely Affected

Disclaimer:
Permission for use of this questionnaire was granted by Dr. Gregory Boyer, PhD
