# Supplementary material for: Pharm-MD; an open-label, randomized controlled, phase II study to evaluate the efficacy of a pharmacist-managed diabetes clinic in high-risk diabetes patients – study protocol for a randomized controlled trial
Source: Trials. 2018 Aug 24;19:458. doi: 10.1186/s13063-018-2836-8 (PMC6109355; doi:10.1186/s13063-018-2836-8)
Supplement: Supplementary file 1 — Institutional Review Board (IRB)-approved informed consent; outcome letter with IRB approval; grant award letter; pharmacy visit template; standard of care (SOC) appointment card; standard of care + pharmacist-managed diabetes clinic (SOC + PMDC) appointment card; pharmacy appointment card; Diabetes-39 questionnaire. (ZIP 443 kb) [file 13063_2018_2836_MOESM1_ESM.zip › Patient recruitment letterR1.pdf]

Beaumont

IRB# 2017-494

Pharm-MD; an Open-Label, Randomized Controlled Phase II Study to Evaluate the Efficacy of a Pharmacist Managed Diabetes Clinic in High-Risk Diabetes patients

Name, Address:
Dear (name)

You are receiving this invitation because you have been getting medical care at the Beaumont Hospital, Royal Oak Internal Medicine Resident Clinic and have a diagnosis of Type 2 Diabetes with a hemoglobin A1c greater than or equal to 9. I am writing to inform you about a new research study that may be of interest to you.

The purpose of the Pharm-MD research study is to investigate the efficacy of a pharmacist managed diabetes clinic in helping to control your diabetes and improve your quality of life. If you decide to take part in this study, you will be assigned to one of two groups. One group will have scheduled visits with their primary care physician, and the second group will have scheduled visits with the pharmacist at Beaumont Hospital, Royal Oak, in addition to the visits with their primary care physician. Once you are assigned to a group, it will not be possible to switch groups for the duration of the study. You will be compensated $15 per visit, and the study will take one year to complete.

Taking part in this research study is completely voluntary. Your care at Beaumont Hospital, Royal Oak will not be affected in any way if you choose not to participate.

How can I learn more about taking part in this research study?

You can contact the study coordinator, Sandy Baker, at 248-551-1841. If you need to leave a message, please speak clearly indicating: your name, your phone number, and the best time to reach you.

If you do not respond to this letter within two weeks, a member of our study team may contact you by telephone to see if you are interested. Thank you for considering this research study.

Sincerely,

Alexandra Halalau, MD

Site Principal Investigator

IRB NUMBER: 2017-494
IRB APPROVAL DATE: 02/06/2018
