# Supplementary material for: Pharm-MD; an open-label, randomized controlled, phase II study to evaluate the efficacy of a pharmacist-managed diabetes clinic in high-risk diabetes patients – study protocol for a randomized controlled trial
Source: Trials. 2018 Aug 24;19:458. doi: 10.1186/s13063-018-2836-8 (PMC6109355; doi:10.1186/s13063-018-2836-8)
Supplement: Supplementary file 1 — Institutional Review Board (IRB)-approved informed consent; outcome letter with IRB approval; grant award letter; pharmacy visit template; standard of care (SOC) appointment card; standard of care + pharmacist-managed diabetes clinic (SOC + PMDC) appointment card; pharmacy appointment card; Diabetes-39 questionnaire. (ZIP 443 kb) [file 13063_2018_2836_MOESM1_ESM.zip › PMDC visit template (1)R1.docx]

**High-Risk Pharmacist-Managed Diabetes Clinic**

**BEAUMONT HEALTH SYSTEM OUTPATIENT CLINICS**

**3535 W. THIRTEEN MILE ROAD, LOWER LEVEL 47**

**ROYAL OAK, MI 48073**

**248-551-2031**

**Pharmacist Progress Note**

**Date/Time:** @TD@ / @NOW@

Patient is a @AGE@ @SEX@ who was seen for diabetes initial visit in the High-Risk Pharmacist-Managed Diabetes Clinic.

Referring Physician:

PCP Physician: @PCP@

Subjective:

Diabetes

**Blood Glucose:** Patient presents with {log} {meter} {no log or meter}. Average blood sugars noted below for the date range:***

Fasting: (average:*** ; range:***)

Lunch:(average:*** ; range:*** )

Dinner: (average:*** ; range:*** )

Bedtime: (average:*** ; range:*** )

**Hypoglycemia:**

Patient experienced {NUMBERS 1-12:10} episodes of hypoglycemia {Time; hourly/daily/weekly:11039}. Hypoglycemic episodes were symptomatic at {Numbers; 0-100:15068}.  The cause of the episodes are ******* and treated with *******.

Hypertension Management

Home BP readings:

Caffeine intake:

Pain assessment:

Smoking status:

Patient {reports} {denies} dizziness, lightheadedness, shortness of breath, chest pain, headache, or edema. Patient {does} {does not} use medications that may worsen hypertension, including ***.

Lifestyle

DIET:

Breakfast:

Lunch:

Dinner:

Snacks:

EXERCISE:

Medication Adherence/Cost:

Patient reports {poor/fair/good/excellent} adherence to prescribed medications with missed doses occurring {Daily/Weekly/Monthly/Rarely Ever}. Regarding adherence {Affordability/General Forgetfulness/Lack of Understanding} appear to be the primary factors involved. *** insurance is the primary source of paying for medications with {no/little/moderate/significant} difficulty affording medications.

**Objective:**

Vital Signs:

**Weight: *****

**Blood Pressure: *****

Laboratory Results:

@LAST3LAB(HGBA1C:3,BUN:3,CREAT:3,GFRNONAFR:3,GFRAFR:3,cholesterol:3,LDL:3,HDL:3,triglyceride:3,lipid:3,MCALBCRERAT:3)@

Kidney Screening: {Up to date OR due} (date of last:***)

Foot Exam: {Up to date OR due} (date of last:***)

Eye Exam: {Up to date OR due} (date of last:***)

ON ACE-I or ARB for HTN or microalbuminuria: Yes/No/Not indicated

On statin therapy: Yes/No/Not indicated

Intensity of statin therapy: {Low/Moderate/High}

Pooled-cohort 10-year risk:

On aspirin therapy: Yes/No/Not indicated

Vaccinations:

Flu vaccine:

Pneumonia:

Hepatitis B:

Tdap:

**Pertinent Diabetes Medications:**

*******

**Insulin Regimen:**

*******

**Patient Education:**

Baseline patient assessment:

Diabetes education topics covered today included:

1. 1. What is diabetes?
2.  Pathophysiology
3.  Signs and symptoms of diabetes
4.  Introduction to diabetes complications
5.  Management of diabetes: individual patient care plan

2.  Monitoring blood glucose and glycemic goals

1.  Self-monitored blood glucose - when to check and why it is important: assess glycemic control and to adjust medications and insulin doses
2.  Hemoglobin A1C goals
3.  Pre- and post-prandial blood glucose goals

3.  Hyperglycemia and hypoglycemia

1.  Definition
2.  Symptoms
3.  Causes
4.  Treatment

4.  Medications – individualized to patient’s anti-diabetic therapy regimen

1.  Mechanism of action
2.  Administration instructions
3.  Review progressive nature of DM and that treatment may change over time
4.  Adverse effects – monitoring and management

5.  Introduction to Lifestyle Changes

1.  Diet
2.  Review food groups
3.  Portion control with plate method
4.  Exercise
5.  At least 30 min x 5 days a week
6. 

**Patient would benefit from continued education on the following topics at future visits:**

**Assessment/Plan:**

1. 1. Diabetes Mellitus: {Controlled/Uncontrolled/Borderline Controlled}, A1c goal < ***.
2. 2. Hypertension: {Controlled/Uncontrolled/Borderline Controlled}, BP goal < ***.
3. 3. Hyperlipidemia: statin therapy {appropriate/inappropriate}

Follow-up:

**Self-Management Goal:**

**Time Spent:** *** minutes,{Face to Face/Phone} consult.

PATIENT VERBALIZED UNDERSTANDING OF CARE PLAN:  Yes

PATIENT ADVISED TO CALL BACK WITH QUESTIONS, CONCERNS, OR CHANGE IN SYMPTOMS. Yes
