# Supplementary material for: Pharm-MD; an open-label, randomized controlled, phase II study to evaluate the efficacy of a pharmacist-managed diabetes clinic in high-risk diabetes patients – study protocol for a randomized controlled trial
Source: Trials. 2018 Aug 24;19:458. doi: 10.1186/s13063-018-2836-8 (PMC6109355; doi:10.1186/s13063-018-2836-8)
Supplement: Supplementary file 1 — Institutional Review Board (IRB)-approved informed consent; outcome letter with IRB approval; grant award letter; pharmacy visit template; standard of care (SOC) appointment card; standard of care + pharmacist-managed diabetes clinic (SOC + PMDC) appointment card; pharmacy appointment card; Diabetes-39 questionnaire. (ZIP 443 kb) [file 13063_2018_2836_MOESM1_ESM.zip › SOC appointment cardR1.pdf]

IRB NUMBER: 2017-494
IRB APPROVAL DATE: 02/06/2018

PHARM-MD; an Open-Label, Randomized Controlled Phase II Study to Evaluate the Efficacy of a Pharmacist Managed Diabetes Clinic in High-Risk Diabetes Patients

Principal Investigator: Dr. Alexandra Halalau

STANDARD OF CARE (SOC) Schedule

PARTICIPANT ID NUMBER ______________

PHYSICIAN VISIT 2 DATE: _____________TIME: ___________

PHYSICIAN VISIT 3 DATE: _____________TIME: _____________

If you are unable to make your appointment, please call 248 551-3000 so we can reschedule as soon as possible. Being part of a study is very important. The data we collect from you will help us learn how to better care for our diabetic patients.

THANK YOU FOR PARTICIPATING
