# Supplementary material for: Pharm-MD; an open-label, randomized controlled, phase II study to evaluate the efficacy of a pharmacist-managed diabetes clinic in high-risk diabetes patients – study protocol for a randomized controlled trial
Source: Trials. 2018 Aug 24;19:458. doi: 10.1186/s13063-018-2836-8 (PMC6109355; doi:10.1186/s13063-018-2836-8)
Supplement: Supplementary file 1 — Institutional Review Board (IRB)-approved informed consent; outcome letter with IRB approval; grant award letter; pharmacy visit template; standard of care (SOC) appointment card; standard of care + pharmacist-managed diabetes clinic (SOC + PMDC) appointment card; pharmacy appointment card; Diabetes-39 questionnaire. (ZIP 443 kb) [file 13063_2018_2836_MOESM1_ESM.zip › Information sheet (1)R1.doc]

**Information Sheet**

**Study Title: Pharm-MD; an Open-Label, Randomized Controlled Phase II Study to Evaluate the Efficacy of a Pharmacist Managed Diabetes Clinic in High-Risk Diabetes patients**

#### Principal Investigator: Alexandra Halalau, MD, FACP

**Address: 3601 W 13 Mile Rd, Royal Oak, Michigan, 48073**

**Hospital:** William Beaumont Hospital, Royal Oak

**Purpose:**

You are being asked to be in a research study to compare standard (usual) care with a primary care physician to the standard care plus attending a pharmacist managed diabetes clinic in improving diabetes control and decreasing the risk of a heart attack or stroke because you have a diagnosis of Diabetes mellitus and your hemoglobin A1c is over 9%. This study is being conducted in the Outpatient Clinic at William Beaumont Hospital, Royal Oak.

**Study Procedures:**

If you take part in the study:

- You will be randomly assigned (like “the flip of a coin”) to one of two groups:
  - **Standard of care (SOC) group:** You will be asked to follow up with your own primary care physician every 3 months as recommended in the diabetes guidelines for a total of 3 visits. Today’s visit will be your baseline visit and then, you will have study visits in 3 months and again in 6 months.

**OR**

- **The Pharmacist Managed Diabetes Clinic (SOC+PMDC) group:** You will be asked to participate in a total of 9 study visits (3 visits with your own primary care physician and 6 visits at the pharmacy clinic). Your first visit to the PMDC will be about one week following today’s baseline visit with your primary care physician. The first visit to the PMDC will be 60-90 minutes in length, with follow-up visits that will last 30-45 minutes. The follow-up visits at the PMDC will be at about 1 week, 2 weeks, 3 weeks, and 4 weeks interval from each other. You will see your primary care physician at 3 months. You will have visits at the PMDC at 4 and 5 months and the final study visit with your primary care physician at 6 months.
- You will fill out a quality of life survey after you are assigned to one of the study groups and again at 6 months.
- We will collect information from your medical record at baseline, 6 months and 12 months on your hemoglobin A1c and lipid tests (cholesterol and triglycerides), your blood pressure, compliance with recommended diabetes screening, any emergency department visits or hospitalizations, and how many clinic visits you attended.
- You will be followed in the study for a 12-month period, but your participation in the clinic visits will be over a 6-month period.
- Throughout the study you might be contacted by the study personnel through mail or phone.

**Benefits:**

As a participant in this research study, there may be no direct benefits for you; however, information from this study may benefit other people now or in the near future as we learn how to better manage the growing number of patients with diabetes.

**Risks:**

Beaumont is committed to upholding strict confidentiality in research and all business practices; however, there could be a rare risk of loss of confidentiality. We are very concerned about your privacy and will make every effort to maintain the security of your records.

If you are enrolled in the SOC+PMDC group, you might have a very low risk of hypoglycemia as you will receive more intense education and medication adjustment. This risk will not be higher than in general population that undergoes more intense diabetes management.

**Costs:**

There will be no costs to you for participating in this research study.

**Compensation:**

For taking part in this study you will compensated for your time and inconvenience. For each completed study visit you will receive a $15 gift card that will be handed to you after the completion of each clinic visit. The study allows for 3 visits if you are assigned to the standard of care group and up to 9 visits if you are assigned to the pharmacist managed diabetes clinic group. The $15 gift card will be given to you only if you complete your visit as scheduled or complete your rescheduled visit within 8 days from the initial visit.

**Confidentiality:**

All information collected from you and your health record will be kept securely in a method approved by Beaumont. The data from this study will not be shared with any other party outside Beaumont Hospital.

**Voluntary Participation/Withdrawal:**

Taking part in this study is voluntary. You may choose not to take part in this study, or if you decide to take part, you can change your mind later and withdraw from the study. Your decision will not change any present or future relationships with William Beaumont Hospital or its affiliates. If you are employed by William Beaumont Hospital or its affiliates, as an employee your participation is completely voluntary and will not impact your job in a positive or negative manner. You will not be allowed to change the study group you have been assigned to while the study is running for 12 months.  Once the study is completed you can switch to the other treatment group.

**Questions:**

If you have any questions about this study now or in the future, you may contact Dr. Alexandra Halalau or one of her research team at the following phone number 248-551-3481. If you have questions or concerns about your rights as a research participant, please contact the Institutional Review Board at 248-551-0662. The Institutional Review Board is charged with the oversight of all human participant research conducted at Beaumont facilities.

**Participation:**

By completing the quality of life surveys, the study visits, and allowing us to review your medical record you are agreeing to participate in this study.
